# Knowledge and attitude of secondary school students in Jos, Nigeria on sickle cell disease

**DOI:** 10.11604/pamj.2013.15.127.2712

**Published:** 2013-08-08

**Authors:** Olarewaju Sunday Olakunle, Enwerem Kenneth, Adebimpe Wasiu Olakekan, Olugbenga-Bello Adenike

**Affiliations:** 1 Department of Community Medicine, LAUTECH Teaching Hospital Ogbomosho Nigeria; 2 Managing Partner, Omega-Cares Foundation, Jos, Plateau State, Nigeria; 3 Department of Community Medicine, College of Health Sciences, Osun State University Osogbo Nigeria

**Keywords:** Sickle cell disease, knowledge, attitude, genotype

## Abstract

**Introduction:**

Knowledge about sickle cell disease among youths could constitute an important variable that influences their premarital attitude and behaviour. The study is to determine the knowledge and attitude on Sickle Cell Disease among selected secondary school students in Jos metropolis, Nigeria.

**Methods:**

A cross sectional descriptive study involving 137 Secondary School Students within Jos metropolis selected by a multistage stratified sampling technique, using self administered structured questionnaire. Data were analyzed using SPSS version 17.

**Results:**

A total of 137 students were interviewed, Christians 88%, modal age range 15-20 years (72%) and males (51%). Majority (83.2%) of the respondents were aware of SCDs, as an inherited disorder (80.0%), affecting the red blood cells (83.0%) but only half (54%) knew that the disease can only be diagnosed through blood test. Also, only 59% knew their genotype and 11. 1% claimed AS genotype. More than one fourth (25.5%) had wrong belief that SCD is caused by evil spirit while 76% showed wrong attitude involving stigmatization towards individuals with sickle cell disease.

**Conclusion:**

Comprehensive knowledge about SCD was found to be low despite good awareness among respondents, but only few knew their haemoglobin genotype. If sickle cell disease control strategies must yield any significant results, there is a need to raise awareness about SCD, especially among students in secondary institutions in Nigeria is recommended.

## Introduction

Sickle cell disease is an autosomal recessive genetically transmitted hemoglobinopathy responsible for considerable morbidity and mortality [1]. It is one of the most common hereditary diseases occurring worldwide, which may affect any organ or system of human body. It is an irreversible, manageable health problem predominantly seen amongst various tribes, worldwide. It is found in many parts of the world, particularly in people whose ancestors come from sub-saharan Africa, India, Saudi-Arabia and Mediterranean country.

In Africa three forms of sickle cell disease are present which include sickle cell anaemia (HbSS), sickle cell haemoglobin C (Hb-SC) and sickle cell thalasaemia (Hb-SSthal) [2]. In Nigeria the prevalence of HbSS is 1-3% and it poses a severe burden on the affected individuals and their families [3]. Children born to two parents with sickle cell trait have a 25% chance of having SCD and a 50% chance of having SCT. Therefore, it is highly important for people of reproductive age group to understand the genetics of SCD, know their own blood type, if they carry the S gene choose in advance of selecting partners for future marriages.

The main pathology in SCD is the trapping of sickle shaped red cells in small blood vessels resulting in blockages. This typically manifests as bone pain, which is one of the most distressing symptom in people affected by SCD [4]. The same process can result in other complications including, strokes, bone necrosis, and kidney failure. Some affected persons also have potentially stigmatising signs including jaundice, leg ulcers, and short stature. This is often precipitated by factors such as infection, dehydration, exhaustion and a change in temperature e.t.c which may often warrant hospitalization of the clients. In addition, it is important to note that social and environmental factors are likely to contribute to the pathogenesis of psychopathology in SCD. An important aspect of the social environment is the attitude and perception of non-suffers towards affected persons. Few studies have demonstrated the relevance of stigmatizing attitudes in the lives of children with SCD and the focus mainly is on experiences of young people with SCD [5–6]. Preconception genetic screening and counselling should be the main focus of efforts at controlling SCD in developing countries because screening is relatively cheap and far less invasive that PND. Besides, the psychological and socioeconomic issues at stake are far easier to manage than when a couple must decide on PND and selective abortion. Though this aspect of control of the disease has not been given enough emphasis in Nigeria and other African countries, prevention of the disease through public education, awareness of one's carrier status and genetic counselling regarding reproductive choices is certainly a better ethical and economic option than prevention through PND and selective abortion of affected foetuses.

In addition, senior secondary school students are usually in relationships that may eventually lead to marriage in future, so issue of pre marital screening may be of concern, as this may be affected by existing knowledge and attitude to SCD. This is central to prevention efforts since the disease is preventable. Therefore, understanding knowledge about sickle cell inheritance, its health and reproductive health implications as well as behaviour towards individual with SCD particularly among secondary school students is important regarding limiting the spread of the diseases. The aim of this study was to assess the knowledge and behavior of secondary school students on sickle cell diseases.

## Methods

This was a descriptive cross sectional study of secondary school students within Jos Metropolis. Jos is metropolis in North central Nigeria, with a good spread of both private and public secondary schools, all under the auspices of the State ministry of health. The prevalence of SCD in the two is not known. Only one school in the town was known to have a SCD awareness club. The study focused on senior secondary school students between Class 1 to Class 3. Many of them are in relationships and may be nurturing the ambition of going into marriage in the nearest future.

Multi-stage random sampling was adopted in selecting the students that were interviewed. There are 12 secondary schools in Jos, Plateau State out of which 3 were randomly selected in the first stage of sampling employing simple balloting. There are three classes in each of the school. In the second stage, simple random sampling was also adopted to select one class by balloting in each of the 3 selected schools. There were a total of 330 students in the three selected classes.

The list of students in the selected classes served as sampling frame for the third stage in a systematic random sampling of one in three students on the students register after picking the first by simple random sampling employing simple balloting.. Those who were not in school were replaced by the next person in the sampling frame. In all, a total of 140 students were recruited from 3 schools after sample size calculation was done using Leslie Fischer's formula for calculation of sample size for single proportions. However, only 137 students consented to participate in the study giving a response rate of 97.9%.

Questionnaire was designed to elicit information on socio-demographic characteristics, knowledge, attitude and behavior on sickle cell disease. The opinion of respondents on the prospects of married individuals with sickle cell traits having children with sickle cell disease was also sought. This semi structured questionnaire was pre-tested among senior secondary schools students in Abuja, a town outside Jos and necessary corrections were done thereafter.

Ethical approval to conduct this study was obtained from LAUTECH Teaching Hospital Osogbo ethical review committee and Plateau State Ministry of Education and the local education authority. Informed consent was obtained from each of the students before the study commenced and after explaining the rationale for the study and ethics to them as a group. The questionnaire was self administered in this study using trained research assistants. To avoid confusion, terminologies were explained to respondents during the data collection processes. Data generated with the questionnaire were edited and checked manually for errors and entered into computer for analysis using SPSS version 11.Data was presented as simple frequency tables while association between categorical variables of interest was determined using the Chi squared test at a significance level of less than or equal to 0.05.

## Results

All one hundred and thirty seven respondents returned useful and completely filled questionnaires. The respondent's age ranged between 9 - 25 years with a mean age 17 ± 3 years and a modal age group 15 - 20 years. There were more males 70 (51%) than female 67 (49%).The majority of the respondents were Christians 121 (88%) while the remaining 12 (9%) were Moslem and 4 (3%) practicing other religions (Table 1).

**Table 1:** Socio demographic characteristics of respondents

Variable (n = I37)	Frequency	Percentage
**Age**		
9- I4 years	27	20
15 – 20 years	I03	75
> 20 years	7	5
**Religion**		
Christian	I2I	88
Moslem	I2	9
Others	4	3
**Sex**		
Male	70	51
Female	67	49

Majority of the respondents 113 (97.4%) were aware of sickle cell disease while the remaining three (2.6%) were not aware. Major source of information include health professional 50 (36.5%), Internet 16 (11.1%), friend 19 (13.8%) and family 25 (18.2%). Majority of the respondents i.e 110 (80.0%) knew the disease as an inherited disorder and 81 (51.9%) responded that they knew someone having the disease. In terms of signs and symptoms, 61 (44.5%) do not know any of the signs and symptoms through which one can identify individual with the disease.

Concerning mode of diagnosis, only 2 (1.4%) knew that the disease can only be diagnosed with blood test, 76 (55.5%) mentioned urine test while 59 (43.1%) had no idea of mode of diagnosis. When respondents were asked about chances of each child carrying SCDx when one of the parent has sickle cell trait, only 29 (21.2%) knew correctly that none of the children will carry SCDx. In response to knowledge on preventive measures, 55 (40.2%) mentioned genetic counselling as key method towards occurrence of the disease (Table 2).

**Table 2:** Distribution of knowledge on sickle cell disease

Variables (n=137)	Frequency	Percentage
**Are you aware of SCD**		
Yes	131	95.6
No	6	4.4
**Sources of information (multiple responses)**		
Health professionals	50	36.5
Internet	16	11.7
Friends	19	13.8
Family	25	18.2
**Causes of SCD**		
Acquired	12	8.8
Inherited	110	80.2
Don't know	15	11.0
**Signs and symptoms of SCD**		
Frequent illness	76	55.5
Don't know	61	45.5
**Know someone with SCD**		
Yes	81	9.1
No	56	40.9
**How is SCD diagnosed**		
Blood test	2	1.4
Urine test	76	55.5
Don't know	59	43.1
**Chances of each child carrying SCD when one of the parents have SC trait**		
None of the children	29	21.2
All the children	3	2.2
Half of the children	43	31.3
Quarter of the children	36	26.2
Don't know	26	19.0
**Chances of each child carrying SCD when both parents have SC trait**		
None of the children	1	0.8
All the children	78	57.0
Half of the children	20	14.6
Quarter of the children	12	87.6
Don't know	26	19.0
**Measures of preventive measures on SCD**		
Genetic counselling	55	40.0
Don't know	82	60.0

In terms of behaviour towards SCDs, 119 (89.6%) believed that someone should know his/her genotype but only 81 (59.2%) knew their genotype. Among those who claim to know their genotype, 48 (59.2%) AA, 9 (11.1) AS while 24 (29.6%) had other combinations. In response to importance question on if genotype will influence the decision of partners in getting married, 59 (43.1%) considered it as an important factor while 68 (66.9%) regarded it as not important. Thirty one (22.3%) will not go ahead marring an individual discovered to have SCD. When asked about what should be done by couple when they discover that their genotype predispose them to having children with SCD, 32 (23.4%) wants the relationship discontinued, 1 (0.7%) wants the concerned couple to carry on with their relationship and dam the consequences, 79 (51.9%) wanted the couples to seek genetic counselling and make informed decision while the remaining 33 (24.1%) had no idea (Table 3).

**Table 3:** Behavior to someone with sickle cell diseases

Variable (n=137)	Frequency	Percentage
**Do you think everybody should know their genotype**		
Yes	119	86.9
No	18	13.1
**Do you know your genotype**		
Yes	81	59.2
No	56	40.8
**If yes, what is your genotype**		
AA	48	59.2
AS	9	11.1
SS	24	29.6
**Will partner genotype influence decision to marry him or her**		
Yes	59	43.1
No	68	66.9
**If your partner have SCD, would you go ahead to marry him or her**		
Yes	31	22.3
No	106	77.7
**What should be done by couple when they discover that their genotype predispose them to having children with SCD**		
Discontinue their relationship	32	23.4
Continue with their relationship and damn the consequences	1	0.7
Seek generic counselling and make informed consent	71	51,9
Don't know	33	24.1

## Discussion

Knowledge of the citizenry of a nation about SCD constitutes an important variable that influences the acceptability, practice and success of premarital genetic counselling. The result from our study showed that majority of the student was within the age group of 15- 20yrs, a reflection of the new school curriculum of 6 years in the secondary school as opposed to the previous 5 years.

Majority of the respondents have heard and are aware of sickle cell disease as an inherited genetic disorder. Our finding is in support of another Nigerian study among secondary school students in Abuja, and undergraduate medical students in Lagos where 81.8% and 84% of the respondents claimed to have heard about sickle cell disease. This is however at variance with a study among high school students in Jamaica and another study among adolescents in India where 49% and 46.2% respectively knew the disease is genetically transmitted [7–11].

In Nigeria, SCD and related education is now being taught as part of social studies in secondary schools, thus creating more awareness though this is limited in rural areas. Major source of information include health professional, Internet, friends and family members which was similar to Jamaica study where half of those who knew about the disease also mentioned newspaper, mass media among others. This is possible in this internet era that has made the world a global village, and students are not left behind in these developments. Of the one hundred and thirty seven students, half (51.9%) claimed they knew someone with the disease, showing that the disease is not a rare one [11]. The higher level of awareness of our study population may be due to the fact that our respondents are likely to be exposed to opportunities e.g. mass media which could widen their knowledge base about diseases, most especially the ones that have a genetic basis since the study is done among city dwellers. However, despite high level of awareness, comprehensive knowledge on mode of diagnosis, signs and symptoms as well as preventive measures is low hence the need for health workers to work closely with school teachers in promoting awareness among secondary school students. These deficiencies need to be corrected and rectified. Preconception genetic screening and counselling should be the main focus of efforts at controlling SCD in developing countries because screening is relatively cheap and far less invasive that Pre-natal diagnosis (PND). Besides, the psychological and socioeconomic issues at stake are far easier to manage than when a couple must decide on PND and selective abortion. Though this aspect of control of the disease has not been given enough emphasis in Nigeria and other African countries, prevention of the disease through public education, awareness of one's carrier status and genetic counselling regarding reproductive choices is certainly a better ethical and economic option than prevention through PND and selective abortion of affected foetuses. The best approach should be genetic counselling and screening at all levels and age groups. Primary prevention can be achieved through population screening with Hb electrophoresis because of the high carrier rate of HbS in Nigeria. Although it is costly, such a strategy will prove cost effective in the long term and should form part of the basic health services in Nigeria. SCD is an entity of high medical significance including social, clinical, haematological, genetic, biochemical, etc.) due to its high morbidity and mortality rate. The most common clinical problems are painful crises, along with other relevant complications, such as acute splenic sequestration, acute chest syndrome, bacterial infections and stroke. Knowledge of the epidemiology of a disease is important, because it enables an understanding of the distribution and determinants of health and disease processes in human populations [12, 13].

As long as very few people know mode of diagnosis and preventive measures, it may be difficult to control the spread of the genetic anomaly in our population. Apart from lack of comprehensive knowledge, less than half of the respondents do not know their genotype and this is quite surprising particularly in Nigerian secondary school where pre- school screening mandates all genotypic screening. The poor result is not unique, similar study done among university students and secondary school students in same country revealed similar findings [15, 15]. Importantly, the medical and psychosocial aspects of sickle cell disease should be well known by secondary school students because sickle cell disease is a chronic condition that usually brings with it a high degree of suffering, especially in children, sickle cell disease deserves special attention. Study done among health care workers and medical students showed better level of awareness on signs and symptoms of the disease since they interact much more with clients suffering from the disease [9]. The behaviour concerning someone with SCD were diverse among the respondents, more about two-thirds of respondents regarded SCD as not an important factor to be considered before marriage while less than half believed that it is important.

These findings are also similar to those of Treadwell and colleagues which noted that although the majority of the respondents correctly believed that sickle was inherited from parents, a few believed that it was acquired through blood transfusion and was contagious [16]. Others studies have assessed the knowledge of sickle cell disease in some communities and all of them conclude that is there is need to sensitise communities and policy makers about the disease, including its screening and adequate management [17–19]. Therefore, sensitization and continuous education among secondary school students and other stakeholders is therefore of paramount importance in areas with high prevalence of such diseases.

## Conclusion

The findings in this study showed a high level of general awareness about the existence of SCD but comprehensive knowledge about the cause and prevention of SCD was low and associated with vast misconceptions. A large percentage did not see its importance in influencing marital decisions. If sickle cell disease control strategies must yield any significant results, more education about SCD, especially among secondary school students in Nigeria is therefore recommended. The use of persons with SCD as peer educators/counsellors should be explored.
